# A Five-Year Analysis of Antibiotic Resistance Trends among Bacteria Identified in Positive Urine Samples in a Tertiary Care Hospital from Bucharest, Romania

**DOI:** 10.3390/antibiotics13020160

**Published:** 2024-02-06

**Authors:** Alina Maria Borcan, Georgiana Radu, Mădălina Simoiu, Elena Liliana Costea, Alexandru Rafila

**Affiliations:** 1 The National Institute of Infectious Diseases “Prof. Dr. Matei Balș”, 021105 Bucharest, Romania; borcanalinamaria@yahoo.com (A.M.B.); arafila@yahoo.com (A.R.); 2 Faculty of Medicine, The University of Medicine and Pharmacy “Carol Davila”, 050474 Bucharest, Romania; georgianaradu17@gmail.com (G.R.); sumedrea_liliana@yahoo.com (E.L.C.)

**Keywords:** uropathogens, urinary tract infection, antibiotic resistance

## Abstract

The rise of multidrug-resistant bacteria (MDR) has resulted in limited treatment options and poorer outcomes for patients. The objective of this study was to analyze the overall antibiotic resistance trends and distribution for pathogens identified in urine samples at the National Institute of Infectious Diseases “Prof. Dr. Matei Balș” from Bucharest, Romania, over a 5-year period. Antibiotic susceptibility testing was performed using automatic systems and the disk diffusion method. ESBL- and carbapenemases-producing strains were identified using immunochromatography tests, and ROSCO Diagnostica kits were used for definitive confirmation. All results were interpreted according to EUCAST clinical breakpoints. Gram-negative rods (GNR) had overall resistance rates higher than 50% for penicillin and 40% for 3rd- and 4th-generation cephalosporins. *Escherichia coli* resistance to fosfomycin (3%) and nitrofurantoin (2%) remains low, and 33.30% of *E. coli*, 48% of *Klebsiella* spp., and 37% of *Pseudomonas aeruginosa* isolates were multidrug-resistant (MDR). All *Acinetobacter baumannii* isolates were MDR by the last year of the study. For Gram-positive cocci (GPC), 37% of all *Enterococcus faecium* strains and 2% of *Enterococcus faecalis* were vancomycin-resistant (VRE). *E. coli*’s incidence in UTIs’ etiology is on a downward trend. The incidence of *Klebsiella* spp. and GPCs is rising. Antibiotic stewardship strategies should be implemented after carefully considering regional variations in etiology and resistance trends.

## 1. Introduction

Urinary tract infections (UTIs), next to upper respiratory tract infections, are among the most common infections worldwide. They are primarily caused by bacteria and have a significant rate of morbidity and mortality associated, representing huge economic and social burdens. UTIs are infections that occur in all age groups but tend to affect primarily young populations and mostly sexually active women aged 18 to 40. More than 60% of women will experience at least one UTI during their lifetime, and a third of them will suffer a recurrent episode during the first 6 months [1]. Global Health Data Exchange reports show that, from 1990 to 2019, the rates of infection, disability-adjusted life-years, and mortality have increased worldwide, putting a huge financial burden on healthcare systems. Thus, given the difficulties health systems and populations face in managing UTIs, there is a crucial need for continuous efforts towards finding more efficient ways for treating pathogens causing them [2]. Historically, GNRs are the most common microorganisms incriminated in the appearance of UTIs, with *Escherichia coli* causing most cases, followed by *Klebsiella* spp., *Acinetobacter* spp., *Enterobacter* spp., and *Proteus* spp. Aside from GNRs, GPCs such as *Staphylococcus saprophyticus* or *Enterococcus* spp. may cause UTIs [3]. It is very important to make a quick and accurate diagnosis to shorten the course of the disease, as well as to find the most effective treatment to prevent progression toward upper urinary tract and renal impairment. 

UTIs typically require an antibiotic treatment. The tendency to inappropriately administer, as well as self-medicate with antibiotics, has led to increased resistance to many available treatment options [3,4]. Antibiotic resistance has a wide distribution dependent on geographical area, genetic changes in strains, diversity in the use of antibiotics, and antibiotic availability in time. Most of the time, the initial antibiotic therapy is empirical [3]. Moreover, many patients self-medicate with antibiotics, do not follow instructions on the correct administration regimen, or stop taking their medication as soon as the symptoms disappear. All these factors have an additive effect on the natural high susceptibility of developing antibiotic resistance mechanisms that pathogens traditionally involved in the occurrence of urinary tract infections have. Thus, high rates of antibiotic-resistant bacteria have made efficient treatment options more and more unavailable. Hence, studying the particular distribution of microorganisms as UTI causative agents in different areas of the world becomes important in increasing the effectiveness of rational antibiotic usage strategies.

High rates of multidrug-resistant (MDR) bacteria have been observed in recent years in both community- and healthcare-associated infections, leading to very limited treatment options and worse outcomes for patients [5]. In 2017, the World Health Organization published a list of priority pathogens to help prioritize the research and development of new antibacterial agents. The highest on the list are carbapenem-resistant *Acinetobacter baumannii*, carbapenem-resistant *Pseudomonas aeruginosa*, and carbapenem-resistant, 3rd-generation cephalosporin-resistant Enterobacterales [6]. Moreover, the Infectious Disease Society of America has begun to refer to a group of nosocomial pathogens as ”ESKAPE” pathogens, meaning a group of antimicrobial-resistant pathogens associated with nosocomial infection that place a significant burden on healthcare systems. The word ESKAPE is an acronym, meaning *Enterococcus faecium*, *Staphylococcus aureus, Klebsiella pneumoniae*, *Acinetobacter baumannii*, *Pseudomonas aeruginosa*, and *Enterobacter* spp. [7]. All these bacteria, apart from *Staphylococcus aureus*, are part of the top pathogens causing UTIs worldwide.

The aim of this retrospective, observational study was to analyze the distribution and antibiotic resistance profiles to the most commonly prescribed antibiotics of UTIs’ pathogens in the National Institute of Infectious Diseases ”Prof. Dr. Matei Balș” from Bucharest, Romania, from 1 January 2018 to 31 December 2022.

## 2. Results

**Figure 1:**
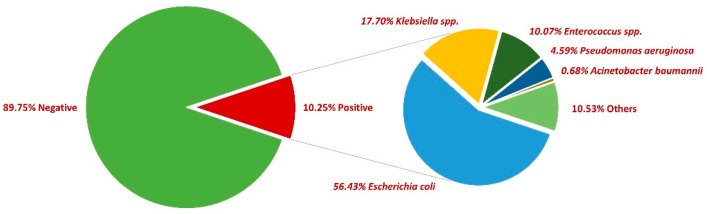
Distribution of bacteria in positive urine cultures between 2018 and 2022.

First, 54,114 midstream urine samples collected between 2018 and 2022 were examined, with a positivity rate of 10.25% (5548). More than two-thirds of positive samples were collected from female patients (73%). The most common pathogen incriminated was *E. coli* (56.43%), followed by *Klebsiella* spp. (17.70%), *Enterococcus* spp. (10.07%), *Pseudomonas aeruginosa* (4.59%), and *Acinetobacter baumannii* (0.68%). In what concerns the rest of the positive urine samples, other bacteria were incriminated, such as *Enterobacter* spp., *Proteus* spp., *Morganella morganii,* and *Staphylococcus saprophyticus*, etc. (Figure 1).

### 2.1. Escherichia coli

*E. coli* was responsible for 56.43% of all UTIs (3131 samples). The highest overall antibiotic resistance was to ampicillin (59%) and trimethoprim/sulfamethoxazole (32%), while the lowest rate of resistance was to gentamicin (8%), fosfomycin (3%), and nitrofurantoin (2%). 

**Figure 2:**
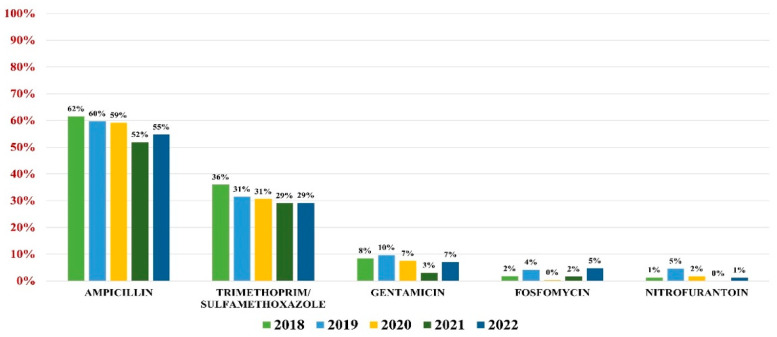
Antibiotics resistance trends for *E. coli*.

In what concerns the overall antibiotic resistance trends, a slight decrease in resistance was observed every year, with the lowest value being recorded in 2021. In 2022, however, the trend resumed its growth (Figure 2).

Of all strains, 13.90% were ESBL producers, while 0.20% were carbapenemases producers. Note that 33.30% of all *E. coli* pathogens were MDR. 

### 2.2. Klebsiella spp.

*Klebsiella* spp. were identified as pathogens for 982 urine samples, representing 17.70% of all positive results; 94% were *Klebsiella pneumonie*, while the rest of them were *K. oxytoca* and *K. aerogenes*. Overall resistance to antibiotics was as follows: amoxicillin/clavulanic acid (44%), ceftazidime (43%), cefepime (40%), gentamicin (31%), trimethoprim/sulfamethoxazole (41%), and colistin (15%).

Of these, 32.90% were ESBL producers and 10.20% were carbapenemases producers. A percentage of 48% were MDR.

**Figure 3:**
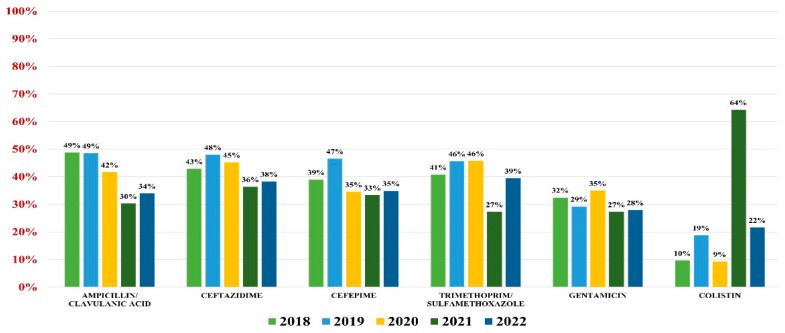
Antibiotics resistance trends for *Klebsiella* spp.

In what concerns the general antibiotic resistance trends, a downward trend in resistance similar to *E. coli* was noted. The trend reached a minimum in 2021 for most antibiotics (excluding colistin) and resumed its growth in 2022 (Figure 3).

### 2.3. Enterococcus spp.

**Figure 4:**
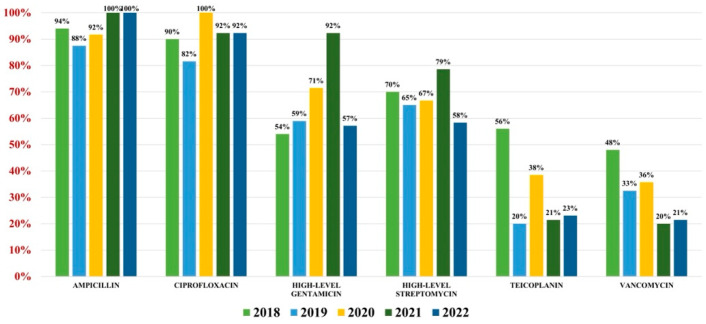
Antibiotics resistance trends for *Enterococcus faecium*.

**Figure 5:**
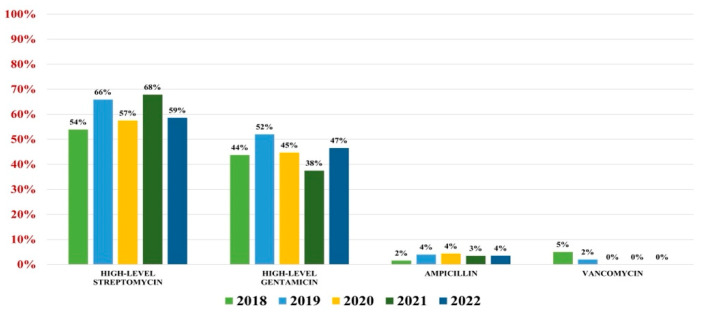
Antibiotics resistance trends for *Enterococcus faecalis*.

Of the 559 *Enterococcus* spp. isolates found, 132 were *Enterococcus faecium* (23.61%) and 427 were *Enterococcus faecalis* (76.39%). Overall antibiotic resistance for *Enterococcus faecium* was as follows: ampicillin (93%), ciprofloxacin (88%), gentamicin-high (62%), streptomycin-high (68%), teicoplanin (36%), tigecycline (3%). In what concerns vancomycin, the overall resistance was 37% (Figure 4). For *Enterococcus faecalis* strains, the antibiotic resistance was as follows: ampicillin (3%), gentamicin-high (46%), streptomycin-high (60%). Note that 2% of strains were VRE (Figure 5).

### 2.4. Pseudomonas aeruginosa

**Figure 6:**
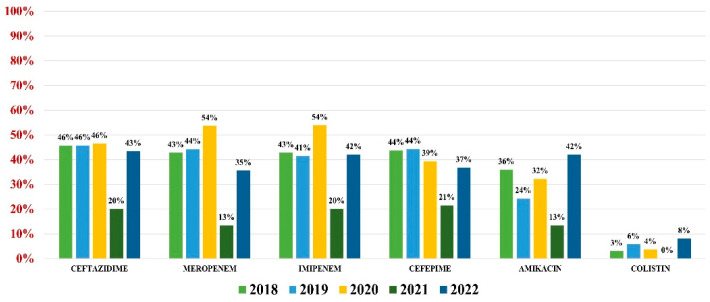
Antibiotics resistance trends for *Pseudomonas aeruginosa*.

*Pseudomonas aeruginosa* was identified in 255 (4.59%) urine samples. Overall resistance to antibiotics was as follows: amikacin (32%), ceftazidime (44%), cefepime (41%), imipenem (42%), meropenem (42%), and colistin (4%) (Figure 6). Note that 37% of all strains were MDR.

### 2.5. Acinetobacter baumannii

**Figure 7:**
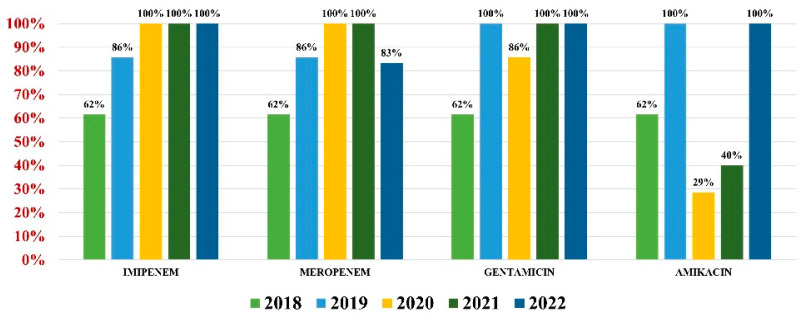
Antibiotics resistance trends for *Acinetobacter baumannii*.

*Acinetobacter baumannii* had an overall antibiotic resistance as follows: gentamicin (84%), ceftazidime (67%), cefepime (67%), imipenem (84%), meropenem (82%), amikacin (66%), and colistin (21%) (Figure 7). Particularly for this pathogen, the percentage of MDR strains has increased from 74% during the first 3 years of our research, to 100% in the last 2 years.

## 3. Discussion

Our study showed a higher incidence of UTIs in female patients compared to males (73% compared to 27%). The reasons for a higher incidence of UTIs in female patients are due to anatomical differences in the lower urinary tract, particularly a shorter and wider urethra, as well as having an external orifice in close proximity to the vagina and anus [8]. The most common cause of UTIs was microorganisms from the Enterobacterales order (GNB). A reason for this is their normal presence in the digestive tract and natural colonization of the perineum, making it easy to contaminate the urethra. Moreover, more than half of all UTIs were caused by *Escherichia coli*. This percentage is lower compared to historically reported prevalences of 80–90%. In other parts of the world, the prevalence ranges from below 50% to 90%: 50–80% in Asia (58% in Saudi Arabia, 70% in India, 75.3% in Turkey, 65.9% in South Korea, 74.8% in Bangladesh), 60–90% in Europe, 64.50% in Portugal, 75–90% in the United States of America, 49.80% in Canada, and 76.60% in Brazil [3,9]. 

*Klebsiella* spp. is the second most prevalent pathogen causing UTIs in many areas around the world [3]. However, several studies found other bacteria, such as GPC, to have a higher prevalence rate [10,11,12]. Its high involvement in the pathology of UTIs is of particular importance as it is very susceptible to the accumulation of antibiotic resistance mechanisms in the context of being continuously exposed to antibacterial agents, as well as other drug-resistant pathogens in healthcare-associated environments [13]. Treatment options for carbapenemases-secreting isolates vary widely with the type of enzyme secreted. Thus, for non-metallo-beta-lactamase (MBL)-producing *K. pneumoniae*, particularly Class A and Class D enzymes, we have the combination between ceftazidime and avibactam, while the combination between meropenem and vaborbactam or imipenem and relebactam can be used particularly for Class A carbapenemases. On the other hand, options for MBL-producing *K. pneumoniae* strains are limited to a combination of ceftazidime, avibactam, and aztreonam, newer antibiotics such as cefiderocol or synergistic combinations (usually colistin-based) [14].

In what concerns antibiotic resistance mechanisms, extended-spectrum beta-lactamase (ESBL)- and carbapenemases-producing bacteria have become a worldwide threat, their incidence rates growing alarmingly in the past decade [15]. Complicated infections with ESBL-producing bacteria may lead to diminished eradication rates, higher recurrence rates, and higher infection-related readmissions for patients. For ESBL *Escherichia coli*, studies from around the world found prevalences ranging from 5% in the United States of America to 11.10% in Canada, 26% in Kuwait, and 33.49% in Saudi Arabia [15,16,17,18,19]. ESBL-producing *K. pneumoniae* bacteria were found to vary in proportion from 9.70% in Canada to 19.50% in France and 58.60% in Ghana [16,20,21]. 

For Gram-positive pathogens, particularly *Enterococcus* spp. and *Staphylococcus* spp., the biggest threat right now is the growing resistance to vancomycin. In the etiology of UTIs, *E. faecalis* isolates are the majority, but most VRE strains are *E. faecium* [22,23,24]. Enterococci are particular bacteria causing UTIs because of their ability to grow in extreme conditions, paired with a high rate of expected resistant phenotypes and multidrug antibiotic resistance [2]. Some populations are predominantly at risk for *Enterococcus faecalis* UTIs, such as young men in whom this pathogen is the second most frequent bacteria incriminated, as well as detected in 47% of polymicrobial infections in men. The reasons for higher susceptibility to *E. faecalis* UTIs in men are still unknown, but some hypotheses are supporting this claim. Firstly, the male prostate is an organ harboring bacteria in small micro-abscesses through bacteria translocation from the intestinal tract which may seed at this level. Secondly, prostate stones and the cavity they reside in may become infected and covered in biofilm [2]. Moreover, there is a significantly higher recurrence rate associated with *E. faecalis* UTIs when compared with *E. coli* [25]. In light of the importance of these pathogens in causing UTIs, it is crucial to monitor antibiotic resistance patterns and implement a rational antibiotic administration strategy in order to delay their development into pan-resistant bacteria, at least until other alternatives become available on a large scale. 

GNRs showed a rather high resistance rate to beta-lactam antibiotics, an expected result in the context of overprescribing, self-medicating, and a dramatic increase in ESBL and carbapenemases secretion for this particular group of bacteria. More than half of *E. coli* isolates were resistant to ampicillin, while for *Klebsiella* spp., the resistance to amoxicillin/clavulanic acid is growing closer to 50%. These results are similar to those from other studies: 55.60% in Spain, 60% in Belgium, 50% in Turkey, and 4% in Poland [3]. In what concerns cephalosporins, resistance to 3rd and 4th classes surpassed 40% for *Klebsiella* spp. and *Pseudomonas aeruginosa* and 60% for *Acinetobacter baumannii*. A 40% proportion of resistance in *Klebsiella* spp. isolates is consistent with other available literature [3]. Considering their efficacy against many bacteria, and the reduced susceptibility to most beta-lactam resistance determinants, carbapenems are considered the most reliable last-resort treatment method [26]. This is why the rapid spread of carbapenem resistance throughout all continents, primarily among GNRs, is a major issue in public health worldwide. Particularly, active surveillance of carbapenem resistance for bacteria such as *Pseudomonas aeruginosa* and *Acinetobacter baumannii* is of crucial importance. In our study, just over 1/3 of *Pseudomonas aeruginosa* isolates, respectively, over 2/3 of *Acinetobacter baumannii* isolates were resistant to carbapenems. Even though the global percentage of carbapenemases-secreting *Pseudomonas aeruginosa* and *Acinetobacter baumannii* isolates varies considerably, the results from our study are consistent with those from other available literature at the moment. Thus, a recent study from the USA reports 10–30% of carbapenem-resistant *Pseudomonas aeruginosa* isolates [27], other countries from Western Europe and Australia report similar rates, while Brazil, Peru, Costa Rica, Russia, Greece, Poland, Iran, and Saudi Arabia have reported percentages higher than 50%. In contrast, the lowest reported rates come from Canada and the Dominican Republic (3.30%, respectively, 8%) [28]. 

There is an estimated annual global incidence of *Acinetobacter baumannii* infections of 1 million cases, of which approximately 50% of strains are MDR, including carbapenems. It is recognized as one of the highest resistant organisms currently encountered by clinicians, and this high level of resistance is the result of a simultaneous and concerted activity of various mechanisms. Carbapenem-resistant *Acinetobacter baumannii* (CRAB) strains tend to be extensively drug-resistant (XDR). Consequently, the incidence of pan-resistant CRAB has begun to rise [29]. The unrecognized presence of *Pseudomonas aeruginosa* and *Acinetobacter baumannii* carbapenem-resistant strains increases the probability of treatment failures and useless expenses on inefficient antibiotic therapies. Moreover, detecting carbapenemases-producing mechanisms in these dangerous pathogens can be challenging for the usual clinical laboratory as it needs molecular genotypic methods or phenotypic methods (ideally both), each having their individual limitations, and can be expensive to procure [27]. 

The burden of multidrug-resistant bacteria is not new, but it has increased during the last two decades. If drastic measures are not implemented quickly, the predicted rate of prevalence for MDR *E. coli* is 77%, and for *K. pneumoniae* it is 58.20% by the year 2030 [30]. In our study, which included 38 *Acinetobacter baumannii* strains, a growing trend of MDR isolates to 100% was observed by the last year. This result appears to be similar to those from the available literature: 90% MDR *Acinetobacter baumannii* strains in the Middle East, 90% MDR in Europe, 85% in Latin America, 80% in Africa, 70% in Asia, and 50% in North America. For this particular pathogen, further research on the use of bacteriophages in the treatment of UTIs may lead to a successful therapy for human treatment [31]. Just like *Acinetobacter baumannii, Pseudomonas aeruginosa* is an important cause of morbidity and mortality in patients with UTIs, despite its reduced incidence (up to 10% in hospital settings) [32,33], due to inherent multiple antibiotic resistance, as well as high susceptibility to develop resistance mechanisms to most classes of antibiotics and form biofilms [34]. In our study, 37% of strains were MDR, similar to results available in the international literature: 34.70% in Portugal [33] and 19% in France [32]. 

Cotrimoxazole (Trimethoprim/Sulfamethoxazole) is one of the most prescribed antibiotic combinations for UTIs. In the last decades, due to an increase in resistance worldwide, its use has been limited and sometimes replaced with other alternatives such as fluoroquinolones. It is also widely available over the counter, thus leading to a high rate of self-medication. Due to these factors, resistance to Cotrimoxazole has seen a dramatic increase, particularly in developing countries (61% in Senegal, 53% in Lebanon, 58% in Turkey). On the other hand, studies from developed countries such as Italy, Canada, Australia, the USA, and Croatia show a lower rate of resistance (varying between 14.50% and 27.10%) [3]. Our study showed comparable results, with more than 1/3 of *E. coli* and *K. pneumoniae* isolates showing resistance to this combination of antibiotics. 

Aminoglycosides are old, very effective antibiotics that have maintained excellent clinical activity against the majority of uropathogens. However, they are mostly used as second-line therapy due to toxicities associated with prolonged administration. In the particular context of growing resistance to first-line therapies (particularly beta-lactams and cotrimoxazole), a renewed interest in prescribing aminoglycosides as first-line therapy for UTIs has emerged, such as single-dose aminoglycoside therapy [35]. This strategy seems to also be of interest in the treatment of pediatric UTIs with ESBL-producing *E. coli* and *K. pneumoniae* [36]. 

Colistin was introduced as a last-resort drug in the context of a dramatic increase in MDR and XDR Enterobacterales, the enormous lack of new, effective antimicrobials, and an increase in carbapenemases-producing pathogens during the last decades. In our study, testing antibiotic susceptibility to colistin was eventually reserved for MDR strains, in an effort to implement an antibiotic stewardship strategy. Moreover, due to the COVID pandemic’s restrictions in 2021, there were periods of time when *Klebsiella* spp. isolates were strictly healthcare-associated; thus, a higher rate of resistance to colistin was expected. Even though resistance rates to colistin seem to be on the rise, particularly for nosocomial pathogens, new ways of using colistin in the treatment of multidrug-resistant infections are in trials, such as combination therapy with other antibiotics or direct intra-vesical administration in order to avoid systemic toxicity [37,38].

Fosfomycin is a relatively old antibiotic, which was somewhat ignored until recently due to the discovery of other antibiotics with a broader spectrum of action. However, fosfomycin is experiencing a comeback in the context of rising antibiotic resistance rates worldwide, especially for treating uncomplicated urinary tract infections [39]. Of course, the fear that bacteria will develop resistance to it arose. Several studies from different countries reported a rapid increase in resistance: Spain (4.40% in 2005 to 11.40% in 2009 for fosfomycin-resistant *E. coli* ESBL isolates), China (34% of KPC carbapenemase-producing *K. pneumoniae* isolates harboring FosA3 gene, suggesting resistance to fosfomycin), and Poland (overall susceptibility of *E. coli* isolates as 77.60% in uncomplicated UTIs). On the other hand, in 2019, the overall resistance of *E. coli* isolates (both ESBL producers and non-ESBL producers) was 2.90%. A recent study from the Czech Republic (Fajfr M. et al., 2020) analyzed the impact of introducing fosfomycin as a treatment for uncomplicated UTIs in the year 2014, showing no increase in resistance levels after the registration and introduction of fosfomycin to UTIs’ treatment regimens (4.40% before the introduction of fosfomycin vs. 3.40% after) [40]. Fosfomycin has been used on a large scale in Romania and our hospital, as a first-line treatment for uncomplicated UTIs. However, the level of resistance for *E. coli* isolates remained low (3%), comparable to that reported by colleagues in the Czech Republic. Another historical wide-spectrum antibiotic recently reintroduced as first-line therapy for uncomplicated lower UTIs is nitrofurantoin. It is particularly efficient against MDR pathogens, as well as prophylaxis for lower-tract UTIs [41,42]. Studies from the available literature report levels of resistance close to 10% for *E. coli* isolates: USA 13.50%, Belgium 10.70%, Pakistan 12.60%, Lithuania 8.30% [43,44,45]. Our study also showed that *E. coli* isolates still have a very high susceptibility rate to nitrofurantoin. 

In general, considering the data from the specialized literature of the last decades, it seems that the pathogens incriminated in the occurrence of UTIs are a constant group of microorganisms, even if their prevalence varies according to geography. The reasons for the extreme differences in resistance and sensitivity in the case of certain bacteria may be due to the varied distribution of resistance genes depending on geography, the consumption of antibiotics, or the lines of therapy for UTIs. The selection of an optimal antibiotic treatment regimen for patients with UTIs depends on an understanding of the regional and temporal patterns of pathogen prevalence, as well as the patterns of resistance to commonly prescribed antibiotics. Thus, continuous surveillance of local pathogens as well as resistance patterns to commonly prescribed antibiotics is recommended. Another important weapon in the fight against multidrug resistance is the implementation of an antibiotic stewardship program with lines of therapy that should be crossed only in the case of resistance to all available antibiotics, at least for UTI treatment, depending on the bacteria incriminated. For the selection of antimicrobial treatments for common infections, cumulative antibiograms performed locally in hospitals are the most effective empirical guide [46]. Starting with 2022, our hospital has been making efforts to implement lines of therapy in the treatment of UTIs according to the level of infection and pathogen. Thus, for lower UTIs, the first line of treatment consists of nitrofurantoin, fosfomycin, trimethoprim/sulfamethoxazole, and ampicillin (for *Enterococcus* spp.), the second line of treatment consists of gentamicin, and the third line consists of carbapenems, vancomycin, and intravenous fosfomycin. For upper UTIs and asymptomatic bacteriuria in the pregnant woman, the first line of therapy offers options such as ampicillin, gentamicin, trimethoprim/sulfamethoxazole, and ampicillin paired with a beta-lactamase inhibitor (particularly sulbactam), the second line of treatment consists of piperacillin/tazobactam and 3rd-generation cephalosporins, and the third line of treatment consists of carbapenems, vancomycin, and intravenous fosfomycin, the same as for lower UTIs. The outcome of this strategy will be analyzed continuously, and it will be adapted regularly according to results in an effort to reduce antimicrobial resistance in our hospital.

## 4. Materials and Methods

### 4.1. Sample Collection and Culture Procedure

The study analyzed all pathogens identified in UTIs from patients of all ages, who were investigated in outpatient or inpatient settings, one-day hospitalization, as well as the Intensive Care Unit. Our hospital mainly treats patients from the southern area of Romania; therefore, the results of our study reflect the antibiotic resistance trends of bacteria in this region. Informed consent was obtained from all individual participants (or legal guardians) included in the study on admission to the hospital, and data were anonymized. Urine samples were collected in sterile containers and arrived in the Microbiology Laboratory within a maximum of 2 h after collection. In the laboratory, samples were inoculated on blood agar, cystine–lactose–electrolyte-deficient (CLED) agar, and Sabouraud Dextrose Agar (BioMerieux, Salt Lake City, Utah) using a calibrated loop (1 µL) under sterile conditions, followed by an 18 ± 2 h incubation period in atmospheric conditions at 35 ± 2 °C. Plates were examined according to laboratory procedures, and samples in which the number of colonies was higher or equal to 100,000 CFU/mL were considered positive for urinary tract infection. Bacteria were identified using the Vitek2 Compact (BioMerieux, Salt Lake City, Utah) and the automated matrix-assisted laser desorption ionization (MALDI) TOF MS (Bruker, Billerica, MA, USA).

### 4.2. Antibiotic Susceptibility Testing

Antibiotic susceptibility testing was performed using the Vitek2 Compact (BioMerieux, Salt Lake City, UT, USA) and MICRONAUT-AM (Bruker, Billerica, Massachusetts) automatic systems. Susceptibility to fosfomycin for *E. coli* isolates was determined using the disc diffusion method, according to the European Committee on Antimicrobial Susceptibility Testing (EUCAST) methodology. NG-Biotech Laboratories immunochromatography tests (Guipry, France) were used for ESBL- and carbapenemases-producing mechanisms identification, and ROSCO Diagnostica (Taastrup, Denmark) kits were used for definitive confirmation. All results were interpreted according to EUCAST clinical breakpoints. All data were organized and interpreted using Microsoft (MS) Excel (version 2019, Redmond, WA, USA).

## 5. Conclusions

UTIs’ etiology has changed in the last years, with *E. coli* becoming less prevalent in favor of other bacteria. The majority of patients with UTIs were women. The prevalence of *Klebsiella* spp. and GPC (particularly *Enterococcus* spp.) is rising. More than 1/3 of all GNRs were MDR. Most of the GNRs displayed high resistance to ampicillin, trimethoprim/sulfamethoxazole, and amoxicillin/clavulanic acid. *E. coli* resistance to fosfomycin and nitrofurantoin remains low. All *Acinetobacter baumannii* strains were MDR by the last year of the study. Surveillance of the characteristics and resistance patterns of local pathogens responsible for UTI etiology is of particular importance. When these parameters are actively monitored, the medical community can recognize resistant strains early on. Rational use of antibiotics is key to reducing the emergence rate of antibiotic-resistant strains.

## Data Availability

Data available on request from the authors.
